# Variation in Quality of Diabetes Care at the Levels of Patient, Physician, and Clinic

**Published:** 2007-12-15

**Authors:** Patrick J O'Connor, William A Rush, Leif I Solberg, Robin R Whitebird, A Lauren Crain, Gestur Davidson, Paul E Johnson, Thomas A Louis

**Affiliations:** HealthPartners Research Foundation; HealthPartners Research Foundation, Minneapolis, Minnesota; HealthPartners Research Foundation, Minneapolis, Minnesota; HealthPartners Research Foundation, Minneapolis, Minnesota; HealthPartners Research Foundation, Minneapolis, Minnesota; University of Minnesota, Minneapolis, Minnesota; University of Minnesota, Minneapolis, Minnesota; The Johns Hopkins University, Baltimore, Maryland

## Abstract

**Introduction:**

We studied variance in glycated hemoglobin (HbA1c) values among adults with diabetes to identify variation in quality of diabetes care at the levels of patient, physician, and clinic, and to identify which levels contribute the most to variation and which variables at each level are related to quality of diabetes care.

**Methods:**

Study subjects were 120 primary care physicians and their 2589 eligible adult patients with diabetes seen at 18 clinics. The dependent variable was HbA1c values recorded in clinical databases. Multivariate hierarchical models were used to partition variation in HbA1c values across the levels of patient, physician, or clinic and to identify significant predictors of HbA1c at each level.

**Results:**

More than 95% of variance in HbA1c values was attributable to the patient level. Much less variance was seen at the physician and clinic level. Inclusion of patient and physician covariates did not substantially change this pattern of results. Intensification of pharmacotherapy (*t* = −7.40, *P* < .01) and  patient age (*t* = 2.10, *P* < .05) were related to favorable change in HbA1c. Physician age, physician specialty, number of diabetes patients per physician, patient comorbidity, and clinic assignment did not predict change in HbA1c value. The overall model with covariates explained 11.8% of change in HbA1c value over time.

**Conclusion:**

These data suggest that most variance in HbA1c values is attributable to patient factors, although physicians play a major role in some patient factors (e.g., intensification of medication). These findings may lead to more effective care-improvement strategies and accountability measures.

## Introduction

Narrowing the wide gap between recommendations on evidence-based diabetes care and actual care delivered to patients (1) would allow tens of thousands of patients in the United States to avoid heart attacks, strokes, amputations, blindness, or end-stage renal disease each year (2). Many strategies to improve diabetes care have been advanced and tested in the last several decades, but overall progress has been modest (3-7) with the exception of some isolated reports of improvement (8-12). Fewer than 20% of adults with type 2 diabetes simultaneously meet evidence-based goals for glycemic control, lipid control, and blood pressure control (13).

Other studies show variation in diabetes care quality at the level of patient (14), physician (15,16), clinic (17), medical group (13), and health plan (18). Hofer et al (19) evaluated variance in glycemic control at two levels of care — the physician and the patient. In those data, the apparent variation in glycemic control at the physician level was explained by simultaneous analysis of patient-level characteristics. Hofer's report did not consider variance at the clinic level, but the findings raise serious doubts about whether variation in HbA1c values across physicians is significant after adjustment for patient characteristics. Krein et al (20) considered variation in diabetes care across three levels — the patient, the physician, and the organization (i.e., clinics and medical groups). They reported that variation at the levels of organization and patient are both greater than variation at the physician level.  

A deeper understanding of variation in glycemic control (as measured by HbA1c values) across organizations, physicians, and patients is important for several reasons. First, if variance in HbA1c values among patients is to be accounted for accurately, then performance measures for physicians and clinics may need to be adjusted to allow for variations in patient characteristics. Second, from the perspective of patient care, accurately mapping variation in glycemic control across levels of care may speed the development of more effective interventions to improve care. For example, if physician specialty is not an important variable, then shifting care of patients from one specialty to another may not improve care. Alternatively, if medication intensification is an important variable, then behavioral or organizational interventions that support medication intensification may be a fruitful way to improve care.

Our 3-year observational cohort study had two purposes: 1) to partition the explainable variance in HbA1c values across the levels of patient, physician, and clinic; and 2) to identify baseline characteristics of patients and physicians that predict control of glycemic levels.

## Methods

### Study design

We analyzed data from adults with diabetes insured by one health plan and receiving care at 18 clinics within one medical group to control for health plan and medical group variation and to simplify the interpretation of results.

### Study setting

This study was conducted at HealthPartners Medical Group (HPMG), a Minnesota multispecialty group practice that provided care to about 180,000 adults at 20 clinics in 1997. The number of patients treated at each clinic ranged from about 6000 to more than 20,000, and nearly all adult primary care was provided by internal medicine or family practice physicians. During the study period, each HPMG clinic had a part-time diabetes nurse educator, and about 30% of adults with diabetes had one or more diabetes educator visits each year. Most of the 10% of adults with diabetes who saw an endocrinologist each year had only one such visit. During the study period, all physicians used the same drug formulary that included insulin, sulfonylurea, and metformin. Primary care physicians (PCPs) provided more than 90% of all diabetes care visits. HPMG adopted an evidence-based diabetes care guideline in 1996 (21,22) and started to use diabetes patient registries in 1997, but active use of guidelines, registries, and other system changes varied from clinic to clinic (8,23). The median HbA1c value of adults with diabetes at HPMG steadily improved from 8.4% in 1994 to 6.9% in 2001.

### Study patients

established diagnosis of diabetes mellitus before January 1, 1995. A diagnosis of diabetes was assigned if the patient 1) had two or more International Statistical Classification of Diseases and Related Health Problems codes (ICD-9 250.xx) codes at inpatient or outpatient encounters in calendar year 1994, or 2) filled a prescription for a diabetes-specific medication (insulin and sulfonylurea) at a health plan pharmacy in calendar year 1994. At this time, nearly all prescriptions written in the study clinics were filled in these pharmacies. This method of identifying adults with diabetes has been previously validated and has estimated sensitivity of 0.91 and positive predictive value of 0.94 (24). We did not attempt to distinguish type 1 from type 2 diabetes in this study, although analyses excluding those who were younger than 40 years and on insulin treatment alone showed similar results.

To be included in the analysis, an adult with diabetes also had to meet the following criteria: 1) complete at least one full year of enrollment at one of the medical group clinics during the 3-year study period; 2) have had at least one HbA1c test in one or more of the three calendar years of the study; and 3) be linkable, based on modal number of primary care visits, to the same PCP in each of the three calendar years of the study. These criteria were developed to ensure adequate nesting of patients by physicians and clinics, and to have adequate data to evaluate the principal dependent variable, glucose control as measured by HbA1c values.

Study patients were evaluated using data collected from January 1, 1995, through December 31, 1997. Patient variables included sex, age, comorbidity score, dates of HbA1c tests, HbA1c values, medication intensification, and study year. These data were extracted from automated clinical and administrative databases for each study year. Characteristics of PCPs (age, sex, and medical specialty) were determined on the basis of the health plan's administrative data. Physicians were linked to clinics on the basis of administrative databases that list the practice site of each physician. Each PCP was assigned to one primary care clinic; physician relocation to a different clinic was limited to about 2% to 3% of physicians during the study period.

### Definition and measurement of variables

All HbA1c assays for which data are included in our analysis were performed at a single centralized, accredited clinical chemistry laboratory that used a standard liquid chromatographic method to measure HbA1c (25). The HbA1c assay has a normal range of 4.5% to 6.1% and a coefficient of variation of 0.058% at an HbA1c value of 8.8%. The assay methodology did not change during the study period, and desktop HbA1c analyzers were not used during the study period.

Each study patient was assigned a modified Charlson comorbidity score in each study year, based on ICD-9 codes assigned at either inpatient or outpatient visits that year (26,27). Two or more ICD-9 codes were required to receive Charlson points for each condition. A Charlson score of zero signifies absence of diagnosis of selected major comorbid conditions at some health care encounter during the index year. The higher the score, the more serious the comorbidity; patients with a score of 3 or higher have a 30% death rate within 24 months. Individuals with no clinic visits for a given year were excluded from the study because they could not be matched to a regular PCP in that year. In this study, no Charlson comorbidity score points were allowed for diabetes.

A diabetes visit was defined as any outpatient visit during the study period with a primary or secondary ICD-9 code of 250.xx. All clinics used the same coding forms within each specialty, and the available diabetes codes were the same for internal medicine and family practice.

The PCP who provided the most diabetes care to each patient in each study year was identified according to the following algorithm. Each outpatient diabetes visit of each patient was classified as being with either a subspecialty physician or with a PCP. If all primary care diabetes visits were with the same PCP in a given calendar year, the patient was linked to that PCP for that year. If more than one PCP saw the patient in a given year, the patient was linked to the PCP whom the patient had visited most often for diabetes that year. In the case of ties between PCPs, the patient was linked to the PCP whom the patient had visited most often regardless of the reason. If this method still resulted in a tie, then "no regular physician" was assigned. If there were no PCP visits for diabetes in a year, "no physician" was assigned.

Medication intensification was measured by counting the number of glucose-lowering classes of drugs the patient was prescribed in each year. Available classes of glucose-lowering drugs were insulin, sulfonylurea, and metformin, the only glucose-lowering agents available on the medical group drug formulary during the years of the study. Patients were coded as 1 for insulin, sulfonylurea, or metformin for each year if they filled a prescription for that drug class in that year; otherwise, they were coded as 0. Medication intensification was defined as either 1) the addition of insulin to the treatment regimen or 2) an increase of at least one drug class to the treatment regimen during a particular calendar year.

### Analysis

For analyses involving a change in HbA1c values over time, the patient was the unit of measurement. We used hierarchical linear modeling with MLwiN software (Centre for Multilevel Modelling, University of Bristol, Bristol, UK) to analyze the data on three levels: patient, physician, and clinic. In the first analytic step, an intercept-only model was constructed with physician identifier and clinic identifier entered for each patient, without other covariates. Intraclass correlation coefficients were estimated for each level of the model; the coeffecients reflect the amount of variance in the dependent variable that is attributable to that level of the analytic model.

In the second analytic step, covariates were added stepwise to the patient level and then to the physician level. Patient-level covariates were age, sex, Charlson comorbidity score (classified as < 3 or ≥ 3), baseline HbA1c, and number of classes of glucose-lowering medications prescribed.  Physician-level covariates included age, sex, and specialty. No clinic-level covariates were entered in the model.

The study was reviewed, approved in advance, and monitored by the HealthPartners Institutional Review Board.

## Results

The number of adults with identified diabetes in 1995 and eligible for inclusion in the analysis each subsequent year was 5432 in 1995, 4835 in 1996, and 4451 in 1997. Of these, 4339 (79.8%) in 1995, 3941 (81.5%) in 1996, and 3767 (84.6%) in 1997 had at least one HbA1c test. Table 1 provides additional information about these patients and compares patients with no HbA1c tests during the study period with patients with one or more HbA1c tests during the study period. Notably, 67% of all patients with diagnosed diabetes mellitus had two or more tests during the study period, and 44% had three or more tests.

Patients linked to a PCP were compared with those not linked to a PCP. These two groups of patients had no differences in sex (47% in both groups were female, *P* = .60), but those linked to a PCP were older (60.4 years vs 55.3 years, *P* < .01), had higher Charlson comorbidity scores (1.75 vs 1.02, *P* < .01), and had higher HbA1c values in the first year (8.3% vs 8.1%, *P* < .01). In addition, those linked to a PCP were more likely to have had an HbA1c test. For 1573 person-years (about 13% of all person-years), no link to a PCP could be made. These person-years were excluded from final analysis after preliminary models indicated that their exclusion had only a minimal effect on the analyses.

Table 2 shows the intercept-only model for the dependent variable of year 1 HbA1c value. Most of the variance in HbA1c value is clearly at the patient level. In the full model with covariates entered at the patient and physician levels, most variance is again at the patient level, with insignificant variance at the physician level, and a small but significant amount of variance at the clinic level. Similar models were constructed with the HbA1c value in year 2 and then in year 3 as the dependent variables, and the same patterns of effect on variance were observed (data not shown). The amount of variance related to the clinic level remained small throughout the study period.

Table 2 also shows the multivariate models comparing change in HbA1c value in intercept-only models and in models that include patient and physician covariates. In these models, only small and insignificant variance components were associated with the physician and clinic levels of the model.

Table 3 presents more detailed data from the full model predicting change in HbA1c. This model explained 11.8% of the variance in change in HbA1c value. Significantly more improvement in HbA1c was noted among the oldest patients and in those who had classes of glucose-lowering medications added during the study period.

Additional models were run using HbA1c test rates, low-density lipoprotein cholesterol test rates, and diabetes eye examination rates as the dependent variables. In each of these analyses, the pattern of results was the same as the results for HbA1c values. In each model, more than 80% of total variance was at the patient level, with much smaller and usually insignificant amounts of variance at the physician and the clinic levels.

## Discussion

Measuring quality of diabetes care at the physician level (28) and at the clinic level (29,30) is a matter of interest for public accountability or pay-for-performance purposes. Our results show little variation in measures of diabetes care at the clinic level and suggest that the variation observed across clinics in other studies may primarily reflect variance from the diabetes care levels of physicians and patients.  

Nonetheless, improving diabetes care in clinics is logical from the management perspective and may lead 1) to changes in physician practice patterns by reducing clinical inertia or 2) to changes in patient variables such as increased adherence to treatment guidelines or readiness to change.

Our statistical results indicate that most variance in measures of diabetes care such as HbA1c values is located at the level of the patient. However, a portion of the variance that the statistical model assigns to the patient level (e.g., variance in HbA1c values related to medication intensification) is strongly influenced by physician attitudes and actions. Because of the intrinsic limitations of the statistical models used in nested analysis, variance at the physician level is underestimated and variance at the patient level is overestimated. In the real world of clinical care, physician and patient engage in a complicated dance that reflects their joint responsibility for care processes and outcomes. Although we recognize that variation in HbA1c values resides at the patient level, the data we present suggest that an accountability approach that assigns most responsibility for care to physicians is reasonable. This pattern of variation suggests that physicians may often need to customize care on the basis of patient age as well as factors such as patient adherence to treatment guidelines, readiness to change, educational level, or health literacy (14,31-34). The pattern of variance also suggests that holding patients accountable for diabetes care results may be a strategy that merits exploration.

Other studies that apply similar analytic approaches were conducted in various health care settings. A study of a geographically dispersed sample of Veterans Administration facilities found facility-level variation (19,20). On the other hand, a study of outpatient care in a limited geographic region showed variation across patients and less at the medical group level (35). For our study, all clinics were nested within one medical group, which may increase homogeneity of clinics and physicians and partially explain why clinics and physicians showed little influence over changes in HbA1c. However, even in homogeneous health care systems, baseline levels of HbA1c varied across clinics and physicians, and clinic and physician use of available diabetes registries and other care improvement tools varies widely (8,12,23).

This study, with its cohort design, power, and accurate pharmaceutical data, supports the hypothesis that medication intensification may be the final common pathway that leads to better glycemic control. For statistical reasons, medication intensification is necessarily measured and analyzed at the patient level, but in clinical practice, medication intensification is usually driven by the physician, with the patient having the final say on whether to act on physician recommendations. Specifically, the strong relationship of medication intensification to variation in HbA1c indicates the difficulty of parsing variance between the patient and physician levels of such models. Although variance related to medication intensification is inexorably assigned to the patient model by the rules of statistics, it is not plausible that patients alone are responsible for medication intensification. Medication intensification is a complex process that involves patient views of disease, patient attitudes toward medications, physician views of disease, physician knowledge and skill in managing various therapeutic agents, experiences of patients and physicians, and important biologic factors such as kidney function, liver function, interactions between drugs, comorbid conditions, and life expectancy. More studies are needed that improve our understanding of patient and physician factors related to medication intensification (35-38). Although we do not report data on lifestyle interventions, other studies clearly show that efforts to improve nutrition, physical activity, and weight are also relevant to the care of most diabetes patients (39).

Our study has a number of limitations. The data were collected at a time when there were fewer medications to treat diabetes than are available now. Patient covariates were limited and did not include factors such as days between visits, adherence to treatment, health literacy, quality of life, or patient readiness to change (14,34). Physician years in practice was not considered, although other studies suggest this variable is not strongly related to diabetes care (12).

We conclude that the great majority of variance in glycemic control resides with the patient. However, variation in a patient's glycemic control is strongly influenced by physician-related factors such as intensification of medications. Improvement strategies that may be effective include those directed to intensification of medications and those directed to patient adherence to medications, among others. The findings also suggest that diabetes quality-of-care measures used for public accountability or pay-for-performance vary at the patient level and that this variation is most likely related to both patient factors and other factors that influence the physician–patient relationship (36,38,40-42).

## Figures and Tables

**Table 1:** Characteristics of Study-Eligible Patients Who Did or Did Not Have at Least One HbA1c Test During the 36-Month Study Period, Multispecialty Health Care Practice, Minnesota, 1995–1997

**Comparison Variables**	**HbA1c Measurement Status**		*P* value

	Measured	Not Measured	
Sex (% female)	53	53	.73
Average patient age, y	59.5	60.4	.10
Charlson comorbidity score^a^	1.73	2.07	.01
Physician (% female)	28	25	.23
Average physician age, y	42.2	43.0	.01
Family physician as doctor (%)	33	35	.39

HbA1c indicates glycated hemogloblin.

a Each patient was assigned a modified Charlson comorbidity score in each study year, on the basis of ICD-9 codes assigned at either inpatient or outpatient visits that year. Higher scores indicate more serious comorbidity. Two or more ICD-9 codes were required to receive Charlson points for each condition (26,27).

**Table 2:** Variance and Change in HbA1c Values at the Levels of Clinic, Physician, and Patient, Multispecialty Health Care Practice, Minnesota, 1995–1997

**Year 1 HbA1c Value (%)**	Intercept-Only Model^a^	***P* value**	Full Model With Covariates	*P* value
Clinic	1.9		2.7	<.05
Physician	2.8	<.05	1.4	
Patient	95.4	<.01	96.0	<.01

**Change in HbA1cvalues, 1995–1997 (%)**	**Intercept-Only Model^a^**	***P* value**	**Full Model With Covariates**	***P* value**
Clinic	<0.1		<0.1	
Physician	0.7		0.8	
Patient	99.3	<.01	99.2	<.01

HbA1c indicates glycated hemogloblin.

a Patient-level covariates were age, sex, Charlson comorbidity score, baseline HbA1c, and number of glucose-lowering medications. Physician-level covariates were age, sex, and specialty. No clinic-level covariates were entered.

**Table 3:** Multivariate Model^a^ Evaluating Relationship of Patient and Physician Covariates to Change in HbA1c Value From 1995 Through 1997, Multispecialty Health Care Practice, Minnesota

Variable	Correlation Coefficient	*t *Statistic
Patient age <65 y	0.093	2.10^b^
Glucose-lowering drug intensification	−0.418	−7.40^c^
Physician specialty	0.057	.55
Patients per physician	−0.002	−1.05
Physician age	−0.001	−.28
Comorbidity score	−0.014	−.76

HbA1c indicates glycated hemogloblin.

a Model R^2^ = 0.118.

b *P *<.05.

c *P *<.01.
